# Nanowired electrodes as outer membrane cytochrome-independent electronic conduit in *Shewanella oneidensis*

**DOI:** 10.1016/j.isci.2022.103853

**Published:** 2022-01-31

**Authors:** David Rehnlund, Guiyeoul Lim, Laura-Alina Philipp, Johannes Gescher

**Affiliations:** 1 Karlsruhe Institute of Technology (KIT), Institute for Applied Biosciences (IAB), Fritz-Haber-Weg 2, 76131, Karlsruhe, Germany; 2 Uppsala University, Department of Chemistry – Ångström, Box 538, 75121, Uppsala, Sweden; 3 Karlsruhe Institute of Technology (KIT), Institute for Biological Interfaces (IBG 1), Hermann-von-Helmholtz-Platz 1, 76344, Eggenstein-Leopoldshafen, Germany; 4 Hamburg University of Technology, Institute of Technical Microbiology, Kasernenstraße 12, 21073 Hamburg, Germany

**Keywords:** Nanotechnology, Biomaterials, Nanostructure

## Abstract

Extracellular electron transfer (EET) from microorganisms to inorganic electrodes is a unique ability of electrochemically active bacteria. Despite rigorous genetic and biochemical screening of the *c*-type cytochromes that make up the EET network, the individual electron transfer steps over the cell membrane remain mostly unresolved. As such, attempts to transplant entire EET chains from native into non-native exoelectrogens have resulted in inferior electron transfer rates. In this study we investigate how nanostructured electrodes can interface with *Shewanella oneidensis* to establish an alternative EET pathway. Improved biocompatibility was observed for densely packed nanostructured surfaces with a low cell-nanowire load distribution during applied external forces. External gravitational forces were needed to establish a bioelectrochemical cell-nanorod interface. Bioelectrochemical analysis showed evidence of nanorod penetration beyond the outer cell membrane of a deletion mutant lacking all outer membrane cytochrome encoding genes that was only electroactive on a nanostructured surface and under external force.

## Highlights

• Exoelectrogenic bacteria were interfaced with nanostructured electrodes
• Ag nanorod arrays with high areal density showed improved biocompatibility
• Alternative extracellular electron transfer via *trans*-membrane nanorod pathway
• Outer membrane cytochrome deletion mutant electroactive on nanostructured interface

## Graphical abstract

**Figure:**
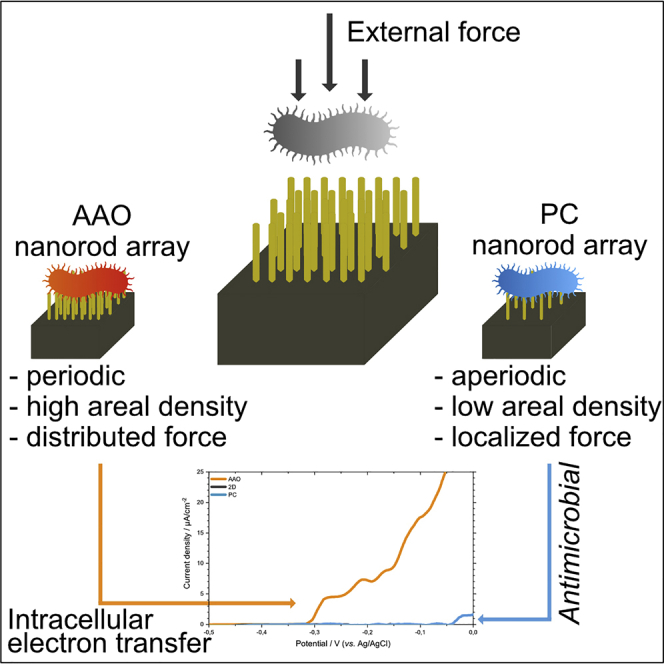

## Introduction

Electrochemically active bacteria have sparked intense scientific interest recently, mainly due to their ability of converting various different organic substrates to higher oxidized compounds of biotechnological interest while producing electrical energy as a by-product in bioelectrochemical systems such as microbial fuel or electrolysis cells (Logan 2009; Logan et al., 2019; Richter et al., 2012). To balance their redox metabolism, respiratory microorganisms require a pathway to transfer electrons to an external terminal electron acceptor. Certain bacteria have evolved a respiratory metabolism using non-soluble metals in oxygen-deprived environments. Bacteria capable of reducing iron- and manganese-based minerals can often achieve this by direct electrical contact between the cell and metal surface (Kumar et al., 2017; Logan 2009; Logan et al., 2019; Richter et al., 2012). Direct electron transfer is made possible by a conductive multiprotein electron transport chain that extends through the cell membrane allowing electron flow outside of the cell. This rather unique ability is most notably expressed in model organisms for dissimilatory iron reduction such as *Shewanella oneidensis* and *Geobacter sulfurreducens*.(Kumar et al., 2017; Logan 2009; Richter et al., 2012). *S. oneidensis* is a particularly versatile organism as it can utilize a wide variety of terminal electron acceptors such as metallic- or carbon-based electrodes as well as various different metal ions (e.g., Fe^3+^, Mn^4+^, U^6+^, and Cr^6+^).(Fredrickson et al., 2008; Gralnick et al., 2006; Hunt et al., 2010; Richter et al., 2012). Extracellular electron transfer (EET) in organisms like *S. oneidensis* stems from a dynamic network of *c*-type cytochromes most of which contain multiple electrochemically active heme groups. The complexity of the EET network is illustrated by the 41 genes that code for *c*-type cytochromes in *S. oneidensis* (Richter et al., 2012). Through rigorous genetic and genomic investigations, a few key proteins have been identified to be crucial for extracellular electron transfers within the organism. CymA is located in the inner membrane and transfers electrons from the menaquinone pool into the periplasm. The periplasm of *S. oneidensis* is too wide (approximately 23.5 nm) for direct electron transfer between membrane-bound cytochromes (i.e., CymA to MtrA) (Beblawy et al., 2018). Electrons are instead transported via soluble periplasmic proteins (e.g., FccA and STC) through the periplasm to the outer membrane. Three proteins (MtrA, MtrB, and MtrC) build an outer membrane-spanning complex that transports electrons through the outer membrane outside the cell where outer membrane cytochromes (e.g., MtrC and OmcA) function as terminal reductases and facilitate the final step of extracellular electron transfer to the electron acceptor. Despite intensive research, a complete picture of the electron transfer network remains elusive (Kumar et al., 2017; Richter et al., 2012). This is mainly due to (1) the multitude of *c*-type cytochromes that are simultaneously expressed and have partially overlapping functions, (2) the lack of specificity of electron transfer reactions between the involved EET proteins, and (3) the commonly used experimental setups that fail in detecting individual electron transfer steps through cytoplasmic membrane, periplasm, and outer membrane. Moreover, experiments aiming at transplanting entire electron transport chains from *S. oneidensis* to *E. coli* revealed that electron transfer can only be partially resolved and the reason for this remains enigmatic (Jensen et al., 2010).

The recent development in nanotechnology has opened up possibilities of integrating nanostructures in biological or medical research, mainly due to their similarity in length scales with cellular components. This new interdisciplinary research field of bionanoelectronics utilizes nanostructured cell interfaces to establish bidirectional communication between cells and electronics (Noy 2011). Trans-membrane nanostructures mainly based on 1D nanomaterials (e.g., nanorods and nanotubes) are introduced into living cells to study internal electrical and biochemical responses to stimuli (Higgins et al., 2020; Robinson et al., 2012; Stewart et al., 2018; VanDersarl et al., 2012; Xie et al., 2012). Spontaneous cell membrane penetration events are rare and a topic of discussion in the field (Higgins et al., 2020; Lee et al., 2014; Robinson et al., 2012; Qing et al., 2014; Xie et al., 2015). Recent modeling reports have shown that cells naturally settling on nanostructured surfaces with dimensions larger than 10 nm in diameter are unlikely to achieve membrane penetration (Xie et al., 2013). The need for external forces to achieve cell-nanorod penetration has led to development of nanostructured intracellular penetration strategies based on well-established membrane disruption techniques such as electroporation, mechanoporation, and optoporation (Higgins et al., 2020; Stewart et al., 2018).

Although the majority of reports focus on eukaryotic cells, there is growing interest in the interaction between prokaryotic cells and high-aspect-ratio nanostructures. This trend is largely driven by the antimicrobial properties (Lin et al., 2018; Tripathy et al., 2017) that nanostructured surfaces have, and there are only a handful of reports on interfacing exoelectrogenic bacteria with nanostructured surfaces for mechanistic studies (Jeong et al., 2013; Jiang et al. 2010, 2013). Bacteria such as *S. oneidensis* and *Sporumosa ovata* have been found to recognize and self-assemble around nanostructured surfaces (Jeong et al., 2013; Sakimoto et al., 2014). Nanostructured electrodes, designed to selectively control the cell-electrode contact, have also been used to study the EET pathway of *S. oneidensis* and *G. sulfurreducens* (Jiang et al. 2010, 2013). Nevertheless, in order to study individual electron transfer steps *in situ*, we need nanomaterials that can interface directly with the microorganism by penetrating the outer membrane. These materials could also allow us to bridge at least the outer membrane of any Gram-negative organism thereby enabling an outer membrane conduit-independent electron transfer pathway. So far, no such studies have been reported to the best of the authors’ knowledge. Nanostructured platforms for intracellular probing are typically based on Si and C nanostructures that require delicate and expensive synthesis (e.g., lithography) (Higgins et al., 2020). Metallic nanostructures can be synthesized using electrodeposition that offers a cost-effective and non-toxic synthesis route with a high degree of scalability in terms of nanostructure dimensions and chemical composition (Edström et al., 2011; Feng et al., 2016; Rehnlund et al., 2015; Walter et al., 2003). Free-standing metal nanostructures can be achieved by template-assisted electrodeposition where the deposition is directed into the pores of nano- or microporous templates. This bottom-up technique utilizes periodic or non-periodic commercial templates based on anodic aluminum oxide and track-etched polymers, respectively (Edström et al., 2011; Hulteen and Martin 1997). The metal nanostructure dimensions are dictated by the template pore dimensions and the coulombic charge of the deposition. Fine control over the deposition rate can easily be achieved through the applied current density. The electrochemical synthesis method has been used to prepare free-standing metallic arrays of Al, Cu, and Ag nanorods (Feng et al., 2016; Oltean et al., 2011; Rehnlund et al., 2015). This well-established synthesis route is highly suited for electrochemically active bacteria since they have a naturally high tolerance for metals, even known antimicrobial noble metals such as Ag and Au (Baudler et al., 2015), making it possible to utilize metal-based nanostructures for intracellular electron detection.

In the present study, we set out to investigate if metallic nanostructured electrodes can be interfaced with the exoelectrogenic bacteria to bridge at least the outer membrane of the organisms. Two different kinds of silver nanostructured electrodes were prepared using template-assisted electrodeposition. The choice of periodic and non-periodic templates (i.e., AAO and PC) allows us to investigate the effect that the nanowire density and areal distribution plays on the bacteria-nanowire interface. The two silver nanorod arrays were investigated and compared based on their biocompatibility and functionality in achieving a stable intracellular interface with native and genetically engineered *S. oneidensis* using centrifugation as the membrane disruption method. This study therefore takes the first steps toward transferring the recent progress in intracellular electron detection used in eukaryotes to the study of electrochemically active bacteria vital for microbial bioelectrochemical systems.

## Results and discussion

### Synthesis of nanostructured electrodes

**Figure 1:**
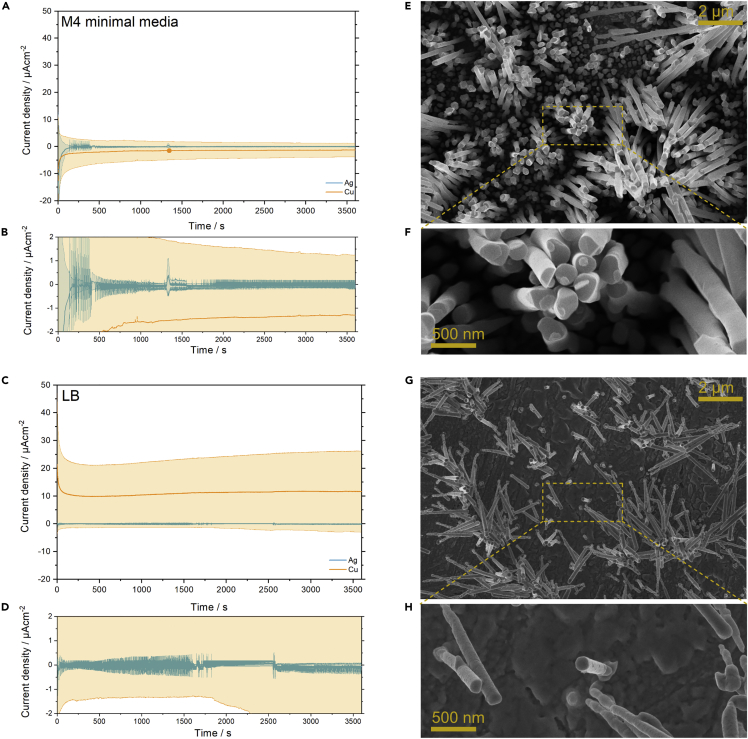
Electrochemical synthesis of nanostructured metal electrodes (A–D) Abiotic chronoamperometry measurements of Ag and Cu planar electrodes in (A and B) M4 minimal media and (C and D) LB media. (B and D) Magnified views of the Ag planar electrode response in both media. Mean current densities are shown with standard deviation error bars. (E–H) Scanning electron microscope (SEM) analysis of pristine silver nanostructured electrodes prepared by template-assisted electrodeposition using nanoporous (E and F) anodic aluminum oxide (AAO) and (G and H) polycarbonate (PC) membranes. Highlighted regions in (e and g) are magnified in (f and h) to show the individual nanorod dimensions and areal distribution.

The first step toward designing a conductive nanostructured surface suitable for intracellular interrogation of exoelectrogenic microorganisms is to define the electrode and media composition. Copper and silver were selected as the electrode material based on their low electric resistivity and high chemical stability. Both metals were characterized using chronoamperometry at 0 V (versus SHE) in LB and M4 minimal media. As shown in Figures 1A–1D, the copper substrate produced a steady reductive current of about 1.3 μA/cm^2^ in the M4 media, whereas an oxidative current of about 11 μA/cm^2^ was seen in the LB media. The silver substrate showed considerably less electrochemical activity in both media. Besides an initial fluctuation of a few microamperes for the M4 media, the silver electrode showed quite similar results in both media with an average current density of about 100 nA/cm^2^. With the goal of minimizing abiotic electrochemical activity, silver is clearly the best choice of material for developing nanostructured electrodes suitable for nanorod-cell interfacing. The choice of media is less obvious as silver seems quite stable in both chemical environments. However, the yeast extract in the LB medium is known to contain flavins that could function as redox active shuttles and contribute to an increased mediated electron transfer (MET) between the organisms and the nanostructured electrode (Logan et al., 2019). As the aim of the study was to investigate the intracellular direct electron transfer (DET) pathway, it would be best to minimize the effect of MET and therefore the flavin-free medium M4 was selected.

Silver nanostructured electrodes were prepared using template-assisted electrodeposition. Nanoporous templates based on anodic aluminum oxide (AAO) and track-etched polycarbonate (PC) membranes were used to prepare arrays of Ag nanorods. The two different templates were chosen based on their periodic (AAO) and non-periodic (PC) pore distribution and the difference in pore density. Similar pore diameters were, however, selected to discard its effect on the biocompatibility. The resulting nanostructured electrodes were compared based on nanorod dimensions, areal distribution, and interrod distances. The electrodeposition was composed of an initial rapid potentiostatic nucleation step followed by pulsed galvanostatic deposition. This protocol is known to improve the deposition coverage and yield a more homogeneous nanorod growth rate in the narrow template channels (Oltean et al., 2011; Rehnlund et al., 2015). The resulting nanostructured surfaces are shown in Figures 1E–1H. With the AAO template, a densely packed array of well-defined Ag nanorods were observed with an average diameter of 220 nm. The dense distribution of nanorods is also seen in the narrow spacing between nanorods (i.e., 40–70 nm) as well as in the high areal loading of nanorods (i.e., 9.8 nanorods/μm^2^). The PC template produced a more diverse distribution of well-defined cylindrical nanorods with an average diameter of 140 nm and a nanorod density of 1.7 nanorods/μm^2^ (see Table 1). Measurement of interrod distances was complicated by the fact that the PC template does not have perpendicular pores, which causes the resulting nanorods to extend at an angle (<90°) from the surface. This also caused considerable clusters of leaning nanorods to form, a feature likewise observed for the AAO template, albeit to a lesser degree. Nanorod agglomeration is a well-known phenomenon for high-aspect-ratio nanostructures, which is caused by a competition between the nanostructure length, flexibility, and interrod adhesion forces (Pokroy et al., 2009; Rehnlund et al., 2015). An important difference is that underneath the nanorod clusters lies a nanorod-covered surface for AAO, whereas the PC deposition leaves a flat Ag surface underneath the protruding nanorods. The AAO deposition therefore produced a more even nanostructure coverage. A comprehensive summery of the nanorod dimensions and electroactive area of the nanostructured electrode is shown in Table 1. The electroactive area was calculated based on the nanostructured surface area and normalized to 1 cm^2^.

**Table 1:** Dimensions of Ag nanostructures as analyzed from SEM micrographs with Fiji image processing software.

Sample	Nanorod area/μm^2^	Diameter/nm	Nanorods/μm^2^ (pristine)	Nanorods/cell (real)	Nanorods/cell (theoretical)	Electroactive area/cm^2^
AAO	0.037 ± 0.005	220 ± 17	9.8	10	26	10
PC	0.015 ± 0.003	140 ± 15	1.7	1.8	65	8
*S. oneidensis*	0.97 ± 0.18					

### Biocompatibility of Ag nanostructured electrodes with *S. oneidensis*

**Figure 2:**
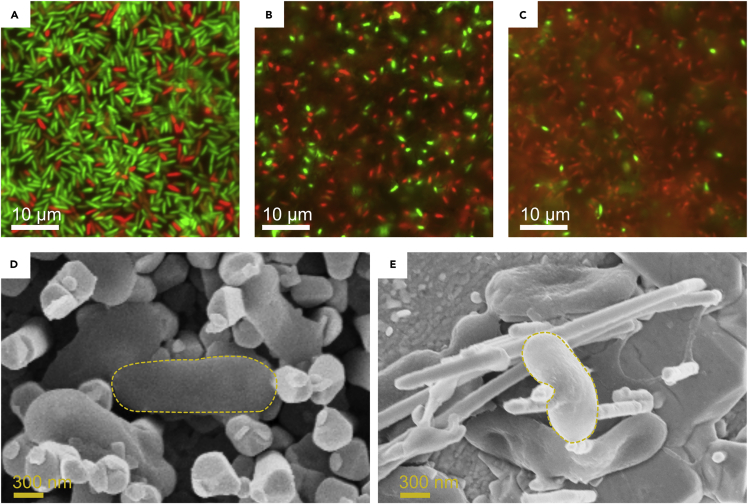
Effect of electrode architecture on the cell viability of wild-type *S. oneidensis* (A–E) Fluorescence microscopy analysis of *S. oneidensis* WT propelled against planar Ag electrodes using centrifugation (i.e., 1,000 × *g*). Live/dead staining was implemented to show the ratio of live (green) to dead (red) cells due to severe cell membrane disruption. The live/dead analysis was performed on (A) 2D planar, (B) AAO, and (C) PC silver electrodes. SEM analysis of (D) AAO and (E) PC nanostructured electrodes after centrifugation with *S. oneidensis* WT. The cell morphology is highlighted in each electron micrograph for clarity.

The biocompatibility of the Ag nanostructured surfaces in regards to *S. oneidensis* cells was evaluated with fluorescence microscopy using commercial live/dead dyes. *S. oneidensis* was cultivated anoxically and applied to the silver electrodes using centrifugation in customized well plates. The similar dimensions of the Ag nanostructures and bacteria such as *S.oneidensi* makes it unlikely that spontaneous membrane-poration would occur. Without external applied force (i.e., without centrifugation) we could not reliably detect cells attached to any of the electrode surfaces (i.e., 2D, PC, or AAO) with fluorescence microscopy. Analysis of the electrode biocompatibility was therefore focused on cells attached with external force. Mechanoporation, using centrifugation as the applying force, was selected as the method of achieving an intracellular bacteria-nanorod interface. In order to minimize cell rupture, a mild treatment of 1,000 × *g* for 5 min was applied to load the cells on the Ag nanostructured surfaces. The centrifugation treatment yielded a stable cell attachment to the nanostructured surfaces as confirmed visually by the nanostructured electrodes becoming pink after the centrifugation step, an indication of cytochrome-rich *S. oneidensis* cells coated on the electrode surface. Also, SEM analysis showed electrodes fully covered by cells, despite the rigorous sample preparation performed prior to electron microscopy analysis. After centrifugation, the cells were stained with the live/dead dye and analyzed by fluorescence microscopy. Figures 2A–2C show the resulting live/dead ratio of *S. oneidensis* WT on both AAO and PC nanostructured surfaces as well as a reference planar (2D) Ag electrode. It is immediately clear that the centrifugation step was not harmful for the organism as most cells survived the treatment on planar Ag surfaces (see Figure 2A). However, the cell viability dropped drastically on the nanostructured surfaces, with the least amount of damage produced by the AAO nanorods. Quantitative analysis of the percentage of living cells revealed that 73.2 ± 7.7%; 39.6 ± 8.7%, and 8.2 ± 0.6% of cells survived on 2D, AAO, and PC surfaces, respectively. This suggests that the AAO nanostructured surface is more biocompatible than its PC equivalent. As seen in the fluorescence image, there seems to be very few cells intact on the PC sample. To help understand the reason for this seemingly antimicrobial property of the PC nanostructures, electron microscopy analysis of the cell-nanorod interface was performed. *S. oneidensis* WT cells were here loaded on AAO and PC nanostructured surfaces (using centrifugation) and then prepared for electron microscopy (see experimental section for EM sample preparation). On the AAO sample, cells are seen to mostly lie evenly on top of the nanorods with some cells squeezed in between nanorods of varying lengths. No evidence of nanorods protruding through the cells could be found. On the PC nanostructures, cells were found to be attached to single and multiple nanorods with evidence that the nanorods are embedded deep within the cell. This fact can be seen in Figure 2E where a highlighted cell is attached to several nanorods and held there above the electrode surface. It therefore seems that *S. oneidensis* cells are more likely to be pierced by PC nanorods than AAO and that this interface leads to the cell membrane puncturing. By comparing SEM micrographs before and after cell loading (i.e., Figures 1E–1H and Figures 2D–2E) we observed that approximately 10 nanorods could connect to one cell for the AAO nanostructures, whereas only approximately 1.8 PC nanorods connected to one cell. Moreover, there was evidence that the PC nanostructures were damaged and disconnected during the centrifugation treatment. In contrast, the AAO nanostructures seemed unaffected by the centrifugation treatment. It is possible that some of the dislodged PC nanostructures caused critical cell damage in the solution phase during the centrifugation, which could explain the low living cell count seen in Figure 2C. Another possibility is that the centripetal force localized on the nanostructured surface exceeded the Young’s modulus of the cell membrane causing it to rupture. The local load distribution of one cell was calculated assuming that the entire cell density was evenly distributed over the electrode surface during the centrifugation step. Loads of 2.4 and 33 kPa were estimated on each cell situated on the AAO and PC nanostructured surfaces, respectively (see supplemental information for further details). The elasticity of *S. oneidensis* biofilms have been found to range between 33 and 38 kPa, as measured by atomic force microscopy in the liquid phase (Kim et al., 2017). Based on the biofilm properties of *S. oneidensis* it would seem plausible that cell membrane rupture could occur on the PC nanostructured surface, whereas it is unlikely on the AAO nanostructured surface. This difference is mainly due to the significantly higher nanorod density found for AAO, leading to cell loads distributed over a larger area.

### Bioelectrochemical analysis of *S. oneidensis* on Ag nanostructured electrodes

**Figure 3:**
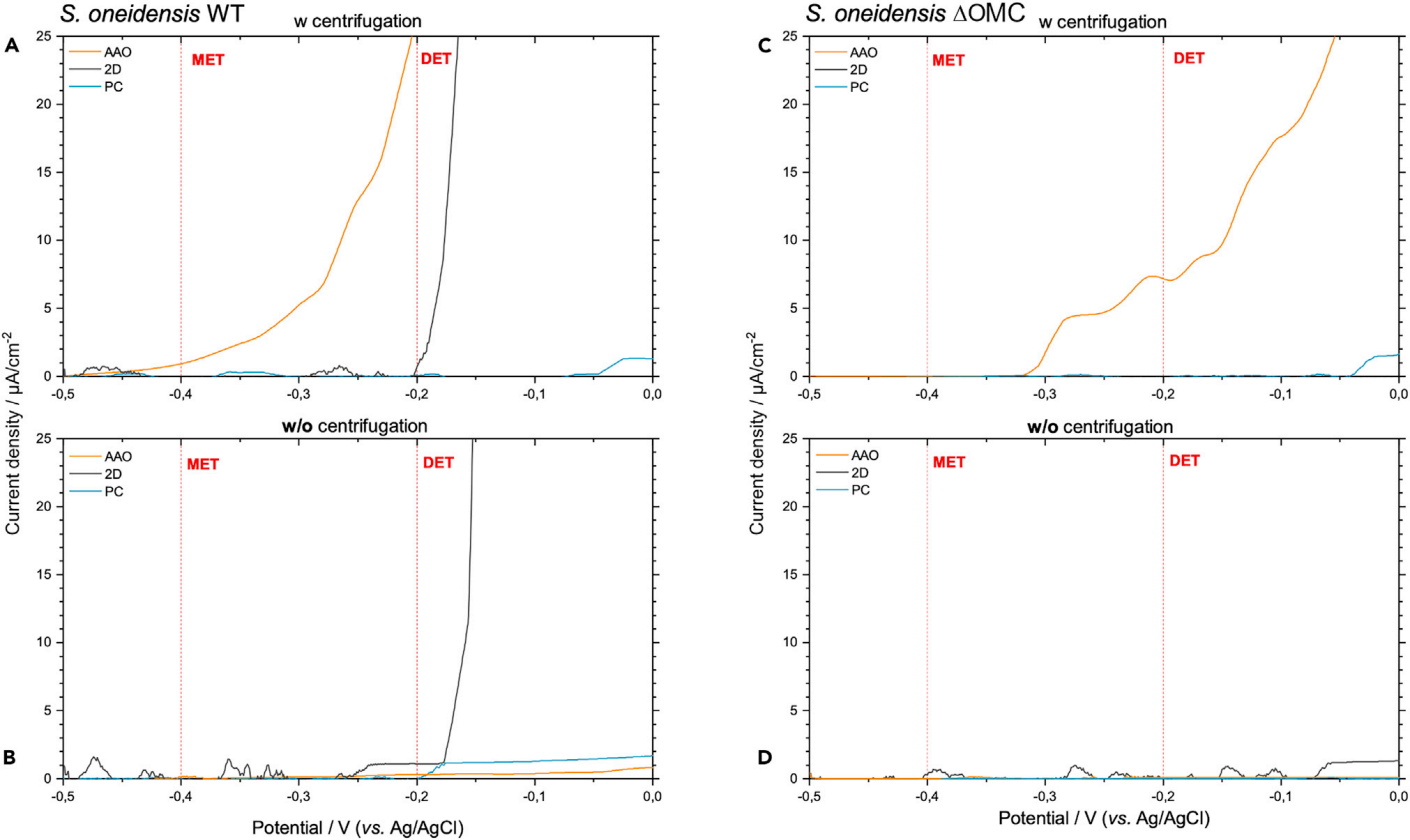
Effect of electrode architecture on the activation potential for *S. oneidensis* strains Linear sweep voltammetry analysis of *S. oneidensis* (A and B) WT and (C and D) ΔOMC deletion mutant (A and C) with and (B and D) without the application of centrifugation prior to the bioelectrochemical analysis. Potential regions where mediative (MET) and direct electron transfer (DET) occur are annotated in each voltammogram. Current densities were normalized to the individual electroactive electrode area.

Next, we investigated the bioelectrochemical activity of *S. oneidensis* WT and a deletion mutant in all five genes encoding outer membrane cytochromes (ΔOMC) on both AAO and PC nanostructured as well as planar Ag (2D). Wild-type *S. oneidensis* can here be regarded as a positive control and is expected to show bioelectrochemical activity on all electrode types. The deletion mutant is a genetically engineered strain where all outer membrane cytochromes are removed leaving it without the ability for extracellular DET (Bücking et al., 2012). First linear sweep voltammetry was implemented to investigate the potential region where microbial oxidation takes place. Both organisms were investigated on the three electrode types with and without centrifugation applied prior to bioelectrochemical analysis (see Figure 3). With centrifugation, the wild-type strain showed a gradual increased oxidative current starting at about −0.45 V that culminated at −0.2 V. The strain showed a direct activation at −0.2 V on 2D Ag electrodes, indicative of mainly DET. In contrast, no significant bioelectrochemical response was observed for the PC nanostructures. Without centrifugation, no significant oxidation took place on either nanostructured electrode, whereas a sharp activation was observed for the 2D electrode. This result is unexpected as the wild-type strain should be able to connect and transfer its metabolic electron pool even without centrifugation, Thus indicating that the centrifugation step can achieve a stable cell loading on the electrode, a feature that takes considerable time without external forces. The ΔOMC strain showed a more selective behavior where only the AAO nanostructured electrode enabled a bioelectrochemical response in the analyzed voltage window. Here an oxidative current response was observed from −0.3 V and steadily increased toward higher potentials. No significant bioelectrochemical signal was observed on the 2D and PC electrodes. Likewise, without applied centrifugation no bioelectrochemical response was observed for either electrode type. Although a lack of bioelectrochemical activity is expected for 2D electrodes with the ΔOMC strain, the results indicate that the AAO nanostructures are uniquely capable of interfacing with the strain to provide a solid electron transfer route that can bypass the lack of outer membrane cytochromes.

**Figure 4:**
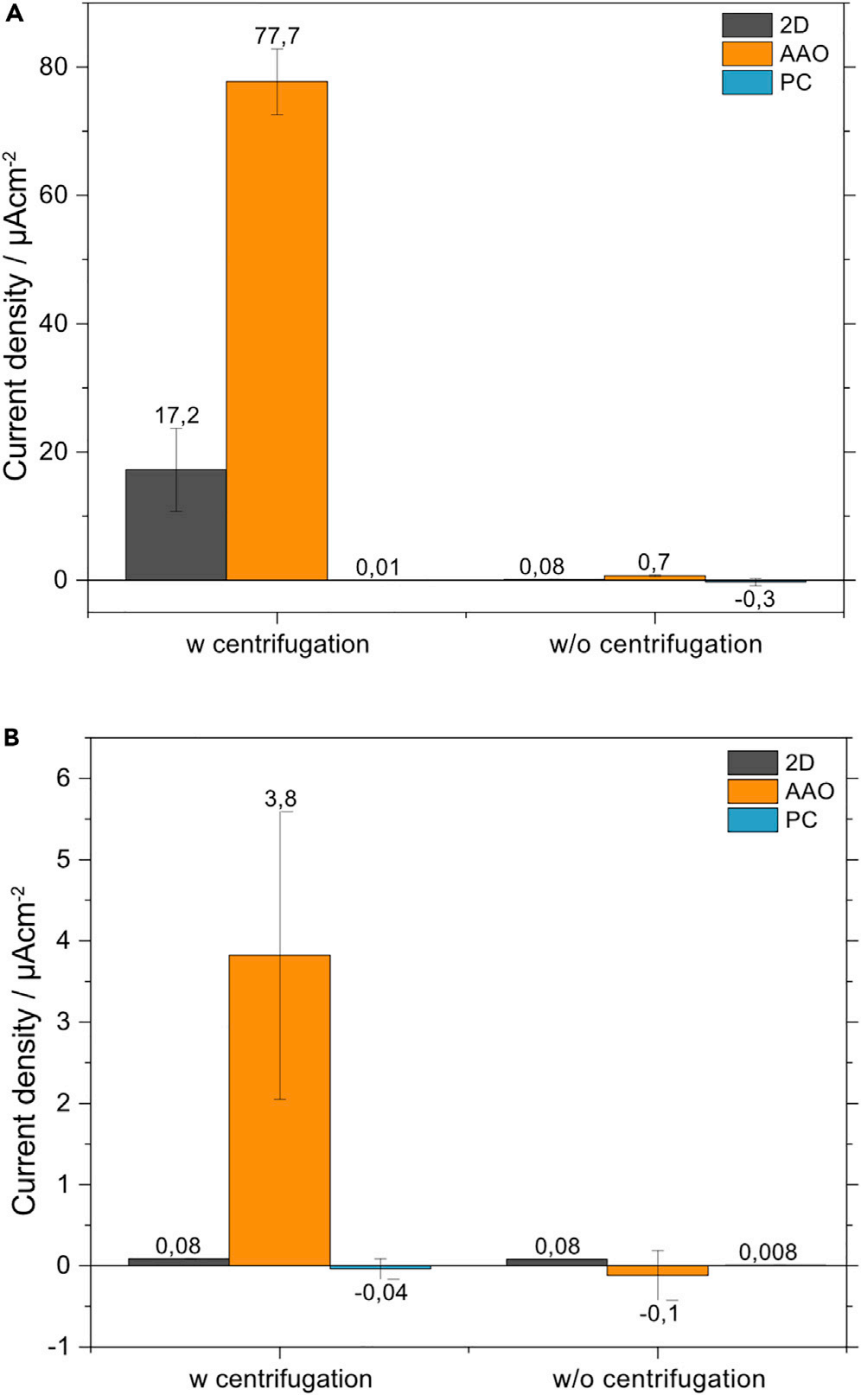
Electrochemical performance of *S. oneidensis* strains (A and B) Chronoamperometry analysis of *S. oneidensis* (A) WT and (B) ΔOMC deletion mutant was performed at −0.2 V (versus Ag/AgCl) with and without the application of centrifugation prior to the bioelectrochemical analysis. The current response was recorded for both strains using 2D (gray), AAO (orange), and PC (blue) Ag electrodes. Statistical analysis of all experiments shows the mean current density (with SD error bars). Current densities were normalized to the individual electroactive electrode area.

After establishing that *S. oneidensis* can be interfaced with the nanostructured electrodes we wanted to further investigate electron transfer of the WT and ΔOMC strain. Chronoamperometry was therefore implemented and set to probe the current response at -0.2 V (versus Ag/AgCl), which corresponds to 0 V (versus SHE). For the WT strain after centrifugation, an initial spike followed by a gradual current density decline was observed on the AAO and 2D electrodes with an average current density of 77.7 ± 5.1 and 17.2 ± 6.5 μA/cm^2^, respectively (see Figures 4A, S2A, and S2B). The PC nanostructured electrode showed comparatively no current response throughout the measurement with an average current density of 0.012 ± 0.001 μA/cm^2^, in agreement with the linear sweep voltammetry results. As expected, without centrifugation no significant current response was observed for either electrode type. The ΔOMC strain showed a similar current response to the WT strain after centrifugation on the AAO nanostructured electrode, albeit with considerably reduced current density (3.8 ± 1.8 μA/cm^2^) (see Figures 4B, S2C, and S2D). In contrast, essentially no current response was observed on the PC and 2D electrode with centrifugation. Without centrifugation, all electrodes showed background level current densities, in agreement with the WT strain results. It can therefore be concluded that an external force (i.e., centrifugation) is needed to achieve a stable cell-electrode connection for DET. The current response observed for the WT strain on 2D and AAO as well as the ΔOMC strain on AAO is typically observed for exoelectrogenic microorganisms in bioelectrochemical systems (see Figure S2). The organisms start fully charged with a fully reduced quinone and cytochrome pool. As the potential of DET is supplied, the organisms can quickly discharge leading to a rapid current response that decays with time as either new organisms are attracted to the electrode surface or existing organisms connected to the electrode produce a steady current through anaerobic respiration. The latter is more likely in the case of forced cell-nanorod integration as shown in Figure 3C. In conclusion, of the two nanostructured surfaces investigated, only the AAO platform resulted in a stable cell-nanorod connection for both the WT and ΔOMC strain. These results are in good agreement with the live/dead fluorescence microscopy results presented in Figure 2.

## Conclusions

Silver nanorod arrays were synthesized using template-assisted electrodeposition with periodic (AAO) and aperiodic (PC) commercial nanoporous templates. The resulting nanostructured electrodes obtained varied nanorod dimension, geometry, and distribution. Densely packed Ag nanorod arrays were obtained with the AAO templates, whereas the PC template yielded a sparser distribution of high-aspect-ratio nanorods. Using centrifugation as external force, *S. oneidensis* cells were attached to the nanostructured surface. This process led to decreased cell viability compared with flat Ag surfaces, where PC showed the most antimicrobial properties. Comparatively, the AAO nanostructured surface maintained a stable biocompatibility with about 40% of cells surviving the treatment. SEM imaging revealed a close interaction between cells and the nanostructured surface with evidence of cells being pierced by PC nanorods, while situated on top of the AAO nanorods. A feature that is represented in the low number of nanorods connected to each cell for PC, which caused an increased localized load distribution on each cell for the PC nanostructured surface compared with the AAO. The superior cell viability on AAO nanostructures is likely caused by the large number of nanorods connected to each cell leading to a low localized cell load during centrifugation.

Bioelectrochemical analysis of the nanostructured electrodes showed a distinct oxidation current coming from *S. oneidensis* WT on the AAO electrode, similar to the response on 2D Ag electrodes. Effective cell-nanorod connections were observed in the MET potential region with dramatic current increase in the DET potential region. Analogous with the poor cell viability, no significant bioelectrochemical activity was observed on the PC nanostructured surface. Thus, there seems to be a strong correlation between cell viability during cell-nanorod interfacing and its bioelectrochemical activity. Studies on the *S. oneidensis* deletion mutant ΔOMC revealed a selective bioelectrochemical response for the AAO electrode with activation taking place at slightly higher potentials compared with the WT strain (i.e., 150 mV). The centrifugation step was also found to be crucial for establishing a strong cell-nanorod interaction as background level currents were observed without centrifugation prior to the bioelectrochemical analysis.

In conclusion, interfacing electroactive bacteria with conductive nanostructured electrodes can be achieved using centrifugation as external force. A strong correlation between the nanostructured geometry and cell viability was observed with improved biocompatibility for highly ordered and densely packed nanorod arrays. Evidence of nanowire penetration beyond the outer cell membrane of *S. oneidensis* was found for an outer membrane cytochrome deletion mutant that was only electroactive on AAO nanostructured surfaces and under external force. This early development of intracellular nanostructured probes for electroactive bacteria could provides an important tool for the ongoing research into transplanting entire EET chains into non-native exoelectrogens and the overall understanding of the EET pathway. In this pursuit, further method development will be needed to actively connect as many living cells as possible to the nanostructured electrode, which can be achieved by, e.g., reducing the nanostructure dimensions and increasing the nanorods/cell coverage to create a more disperse load distribution over the cell membrane. The application of this new method is especially interesting for microbial bioelectrosynthesis technologies as we can now basically connect any microorganism to a nanostructured electrode and feed it electric current, the cheapest and most sustainable electron donor, for production of various important biofuels and platform chemicals.

### Limitations of the study

This study takes the first step to integrating intracellular electron detection in exoelectrogenic bacteria using nanostructured electrodes. Although the Ag nanowired arrays based on AAO templates showed superior biocompatibility and electrochemical response, the use of 220-nm-wide nanorods was not ideal given the similar dimensions of *S. oneidensis*. Future studies will focus on developing nanowired arrays with sub-100-nm diameters, while maintaining a high areal density, to further improve the signal-to-noise ratio. The synthesis could thereby be aided by the use of templates with a metallic thin film sputtered on one side for electrical contact and more homogeneous growth rate during electrodeposition.

## STAR★Methods

### Key resources table

**Table:** 

RESOURCE or REAGENT	SOURCE	IDENTIFIER
**Chemicals,** p**eptides, and** r**ecombinant** p**roteins**		

Tryptone	Carl Roth	Cat# 8952.2
Yeast extract	Carl Roth	Cat# 2904.3
NaCl	Carl Roth	Cat# 9265.1
HEPES	Carl Roth	Cat# HN78.3
Fumarate	Sigma-Aldrich	Cat# F1506
Lactate	Sigma-Aldrich	Cat# 71723-1L
K_2_HPO_4_	Carl Roth	Cat# 6878.1
NaHCO_3_	Carl Roth	Cat# 8551.1
(NH4)_2_SO_4_	Carl Roth	Cat# 3746.1
KH_2_PO_4_	Carl Roth	Cat# P018.2
MgSO_4_	Carl Roth	Cat# T888.2
CaCl_2_	Carl Roth	Cat# 5239.1
Casamino acids	Carl Roth	Cat# AE41.1
Lactate	Sigma-Aldrich	Cat# 71723-1L
CoCl_2_	Sigma-Aldrich	Cat# 449776
CuSO_4_	Sigma-Aldrich	Cat# C1297
H_3_BO_3_	Carl Roth	Cat# 6943.2
FeCl_2_	Sigma-Aldrich	Cat# 44939
MnSO_4_	Carl Roth	Cat# 4487.1
Na_2_EDTA	Carl Roth	Cat# 8043.1
Na_2_MoO_4_	Sigma-Aldrich	Cat# 331058
Na_2_SeO_4_	Sigma-Aldrich	Cat# 71948
NiCl_2_	Sigma-Aldrich	Cat# 654507
ZnSO_4_	Carl Roth	Cat# K301.1
AgNO_3_	Carl Roth	Cat# 9370.1
NH_4_NO_3_	Carl Roth	Cat# X988.2
NaOH	VWR	Cat# 1.064.980.500
Dichloromethane	Carl Roth	Cat# 8424.1
Syto 9	Thermofischer	Cat# L34856
Propidium iodide	Thermofischer	Cat# L34856
Dimethyl sulphoxide	Carl Roth	Cat# 4720.1
Ethanol	VWR	Cat# 85033.460DB
Formaldehyde	Carl Roth	Cat# CP10.1
Ag foil	Chempur	Cat# 904256
Polydimethylsiloxane (PDMS)	Biesterfeld Chemicals	Cat# 5498840000 SYLGARD 184,1,1kg Kit
Polycarbonate membrane (Nuclepore)	Sigma-Aldrich	Cat# WHA110410
Anodic aluminium oxide membrane (Anodisc)	Sigma-Aldrich	Cat# WHA68096022
6-well plate	Sarstedt	Product # 83.3920
Pt mesh	Chempur	Cat# 900338
Ag/AgCl reference electrode	BASi	Product # MF-2052

**Other**		

Potentiostat	BioLogic	Product name SP-200
Centrifuge	Eppendorf	Centrifuge 5810 R
Vinyl Anaerobic chamber	Coy Laboratory Products	Cat # 032714
Potentiostat	BioLogic	Product name : Sensorstat
Fluorescent microscope DM6000	Leica	n/a
Scanning Electron Microscope Leo 1530 Gemini	Zeiss	n/a

**Softwareand** a**lgorithms**		

Fiji	Open source	https://imagej.net/software/fiji/

### Resource availability

#### Lead contact

Further information and requests for resources and reagents should be directed to and will be fulfilled by the lead contact, David Rehnlund (david.rehnlund@kemi.uu.se).

#### Materials availability

No new, unique reagents were developed in this study.

### Experimental model and subject details

#### Cultivation and centrifugation of *S. oneidensis MR-1*

Two strains of *S. oneidensis* were used in the study: *Shewanella oneidensis* MR-1 wild type and a deletion mutant devoid of any outer membrane cytochrome encoding genes labeled ΔOMC.(Bücking et al., 2012). Single colonies of the two *S. oneidensis* strains (WT and ΔOMC) were grown in the absence of antibiotics in Luria Bertani broth (LB) (see Table S1 in supplemental information for composition). All strains were cultivated overnight (approx. 12 hours) in 10 mL of LB at 30°C with 150 rpm agitation. 10 mL of culture was then transferred in 250 mL LB and cultivated over day (approx. 8 hours) at 30°C with 150 rpm agitation. The cells were spun down at 6000 g for 10 minutes and resuspended in 250 mL of LB with 50 mM HEPES, 50 mM fumarate and 50 mM lactate added (see Table S2 in supplemental information for composition). The culture was transferred into a sterile bottle with a gas tight rubber-stopper and incubated with shaking (150 rpm) at room temperature overnight (approx. 12 hours). This step introduced a natural conversion from oxic to anoxic environment as the cultures respiration gradually depleted the oxygen concentration inside the gas tight bottle. Next, the cells were washed three times in M4 medium with 50 mM lactate added using centrifugation at 6000*g* for 10 minutes (see Table S3 in supplemental information for composition). Cells were diluted to an OD_600_ of 1.5 in anoxic M4 medium supplemented with 50 mM lactate and used for the fluorescence microscopy and bioelectrochemical analyses.

Each well was filled with 8 mL of OD_600_ = 1.5 culture (*S. oneidensis* WT or ΔOMC) and either secured in the centrifuge or directly transferred to the anoxic chamber. The well plates were centrifuged (Eppendorf, Centrifuge 5810 R) in a swing bucket rotor at 1000 g for 5 minutes at 4°C. The well plate BESs were then introduced into the anoxic chamber during which the reference and counter electrodes were added to the setup. During this step an additional 3 mL of cell culture was added to ensure that the reference and counter electrodes were well immersed in the medium. Care was taken to minimize the time the cells were exposed to the oxic environment during centrifugation.

### Method details

#### Electrochemical synthesis of Ag nanorods

Nanostructured silver electrodes were prepared by template-assisted electrodeposition using a combination of potentiostatic nucleation and pulsed galvanostatic deposition, based on previous work on Cu and Al nanorod electrodes(Oltean et al., 2011; Valvo et al., 2014). Arrays of free-standing Ag nanorods were grown on silver substrates (Ag foil, Chempur) in an aqueous electrolyte containing 50 mM silver nitrate (AgNO_3_, ROTH) and 100 mM ammonium nitrate (NH_4_NO_3_, ROTH). The silver substrates were first cut into 1 × 1 cm samples and soldered on to a copper wire. Polydimethylsiloxane (PDMS) was applied to the solder joint including its copper wire to insulate the surface and prevent unwanted contact with the electrolyte. The silver substrates were polished and cleaned with ethanol prior to electrodeposition. Aperiodic track-etched polycarbonate (PC) membranes (Nuclepore, Whatman) and periodic anodic aluminum oxide (AAO) membranes (Anodisc, Whatman) were used with pore diameters of 100 and 200 nm, respectively. The two-electrode electrodeposition cell composed of the silver substrate working electrode, an electrolyte-soaked membrane filter (AAO or PC), an electrolyte-soaked glass fiber separator and a silver foil used as counter electrode, all positioned between two plastic plates equipped with silver foil contacts in the mentioned order (see Figure S3 for a schematic representation of the electrodeposition cell). The entire assembly was then compressed and placed vertically in a petri dish containing the electrolyte solution. The electrodeposition protocol included i) an initial nucleation pulse using chronoamperometry at −1,5 V (vs Ag/Ag^+^) for 1 s followed by ii) a rest period at the open circuit potential for 60 s and finally iii) pulsed galvanostatic deposition using chronopotentiometry pulses of 0 and -10 mA/cm^2^ applied for 0.1 s and 0.01 s, respectively. The pulse cycle was then repeated for 20.000 and 10.000 cycles for AAO and PC templates, respectively. A galvanostat/potentiostat (SP-200, Biologic) was used for the electrodeposition procedure. After deposition, removal of the templates was performed by immersing the Ag electrode with attached template in either 0.1 M NaOH or dicholoromethane to dissolve the AAO or PC template, respectively. The now exposed free-standing Ag nanorod array samples were rinsed in water and allowed to dry in air.

#### Bioelectrochemical system setup

Modified 6-well plate (Sarstedt) bioelectrochemical systems (BESs) were prepared to facilitate both bioelectrochemical analysis and centrifugation. The well plate was equipped with three three-electrode cells each containing a silver electrode working electrode, a Pt mesh (Chempur) counter electrode and a Ag/AgCl reference electrode (BASI) (see Figure S4 for a schematic representation of the customized BES). In order to avoid cross-contamination only three of six wells were used per plate. The silver working electrodes were secured in the bottom of the well plate during the centrifugation step and bioelectrochemical analysis. Reference and counter electrodes were connected through the lid which was used to avoid media evaporation during the bioelectrochemical analysis in the anaerobic chamber. For each duplicate experiment, with one type of electrode and strain, one well plate was used.

#### Bioelectrochemical analysis

A multi-step bioelectrochemical analysis of *S. oneidensis* covered Ag electrodes (i.e. 2D, AAO and PC) was performed with and without centrifugation applied prior to the measurement. M4 media supplemented with 50 mM lactate was used during the bioelectrochemical analysis. The analysis was performed in an anaerobic chamber (Coy Laboratory Products) using a multi-channel potentiostat (Sensorstat, Biologic). Once transferred into the anaerobic chamber, a 30 minute pause period was applied to the BES well plates to allow any residual oxygen to deplete. Linear sweep voltammetry (LSV) was first applied by sweeping the potential from −0.5 to 0 V (vs. Ag/AgCl) at a rate of 1 mV/s. Chronoamperometry measurements were then performed at −0.2 V (vs. Ag/AgCl) for 1 hour.

Abiotic electrochemical analysis of copper and silver planar electrodes was performed in the customised BES well plates under anoxic conditions (i.e. inside the anaerobic chamber). Chronoamperometry at −0.2 V (vs. Ag/AgCl) was applied for 1 hour on each electrode type in either LB media or M4 minimal media (see Tables S2 and S3 in the supplemental information for composition).

#### Fluorescence microscopy analysis

The influence of the centrifugation procedure on the cell viability of *S. oneidensis* was investigated using fluorescence microscopy with a commercial live/dead staining kit. Silver nanostructured and planar electrodes were attached to the bottom of a well plate and filled with 5 mL of OD_600_ = 1.5 *S oneidensis* WT culture, as described in the ‘cultivation and centrifugation of S. oneidensis MR-1’ section. Centrifugation at 1000*g* was applied for 5 minutes at 4°C after which the cells were stained and prepared for fluorescence microscopy. The cells were given approximately 30 minutes to settle down on the electrode surface, which was based on the estimated time required for *S. oneidensis* to consume any residual oxygen when introduced into the anaerobic chamber during the bioelectrochemical analysis. Fluorescent dyes comprised of 5 mM of Syto 9 (Thermofisher) and 50 mM of propidium iodide (Thermofisher) were dissolved in dimethyl sulphoxide (Carl Roth) in the dark. 50 μL of the dye mixture was added to each sample and allowed to infiltrate for 15 minutes before fluorescence microscopy analysis. Live/dead analysis was performed by imaging the fluorescent signal of Syto9 (live) and propidium iodide (dead) cells. A motorized fluorescence phase contrast microscope (Leica DM6000) was used for the triplicate analysis of each electrode (i.e. 2D, AAO and PC). Post data analysis was performed with the open source imaging software Fiji.

#### Electron microscopy analysis

Scanning electron microscopy (SEM) was implemented to study the cell-nanorod interactions after centrifugation as well as the pristine nanostructured electrodes using a scanning electron microscope (LEO 1530 Gemini with Schottky field emitter, Zeiss). *S. oneidensis* WT cells were loaded on AAO and PC nanostructured electrodes according to the method presented above in the ‘fluorescence microscopy analysis’ section. A lower cell density culture (i.e. OD_600_ = 0.1) was used to improve the visualisation of individual cells on the nanostructured surfaces. SEM sample preparation was performed by first fixing the cells in a 4% formaldehyde solution dissolved in M4 salt buffer (see Table S3 in the supplemental information for composition) for 1 hour at 4°C. A dilution series in increasing concentrations of ethanol was then applied to remove the water content (i.e. 0, 25 and 50% ethanol) after which the sample was frozen at −80°C overnight and then freeze dried at −80°C and under vacuum for approx. 8 hours. The samples were sputter coated with a 5 nm conductive Pt layer prior to SEM analysis. Post analysis of the SEM images was performed using the open source imaging software Fiji.

#### Calculation of localized load distribution during centrifugation

The load distribution on each cell situated on the Ag nanostructured electrodes during the centrifugation step was calculated based on Equation 1.

Equation 1. Calculation of load distribution.

Equation 1 $$P=\frac{a\times m\times g\times x}{A\times n}$$

Where m is the mass of one cell, a is acceleration of gravity, g is the applied force during centrifugation, x is number of cells stacked on top of each other during centrifugation, A is the nanowire tip area and n is the number of nanorods connected to each cell. The mass of one cell is approx. 10^−15^ kg, acceleration of gravity is 9.8 m/s^2^, the centrifugation force is 1000 g and A was calculated to be 1,54 10^−14^ and 3,80 10^−14^ m^2^ for PC and AAO nanorods, respectively. Quantitative analysis of the number of nanorods connected to each cell was performed with the Fiji software of SEM data resulting in 1.8 and 10 nanorods/cell for PC and AAO nanostructured surfaces, respectively. During centrifugation all cells will be accelerated toward the Ag nanostructured surface leading to multiple cells stacked on top of each other. That number was calculated by dividing the total number of cells in the culture with the area of one *S. oneidensis* WT cell (obtained from SEM data). Approx. 93 cells would push down on each underlying cell attached to the nanostructured surface. The localized load distribution that affect each cell situated on the nanostructured surfaces was calculated to be approx. 33 kPa and 2.4 kPa for the PC and AAO nanostructured electrodes, respectively.

### Quantification and statistical analysis

The data shown in this study is presented as mean ± SD.

## Data Availability

• Original data reported in this paper will be provided upon demand by the lead contact, David Rehnlund (david.rehnlund@kemi.uu.se) • This paper does not report original code • Any additional information required to reanalyze the data reported in this paper is available from the lead contact upon request

## Footnotes

Supplemental information can be found online at https://doi.org/10.1016/j.isci.2022.103853.
