# Supplementary material for: Nanowired electrodes as outer membrane cytochrome-independent electronic conduit in Shewanella oneidensis
Source: iScience. 2022 Jan 31;25(2):103853. doi: 10.1016/j.isci.2022.103853 (PMC8851274; doi:10.1016/j.isci.2022.103853)
Supplement: Document S1. Figures S1–S4 and Tables S1–S3 [file mmc1.pdf]

iScience, Volume *25*

Supplemental information

Nanowired electrodes as outer membrane cytochrome-independent electronic conduit in *Shewanella oneidensis*

David Rehnlund, Guiyeoul Lim, Laura-Alina Philipp, and Johannes Gescher

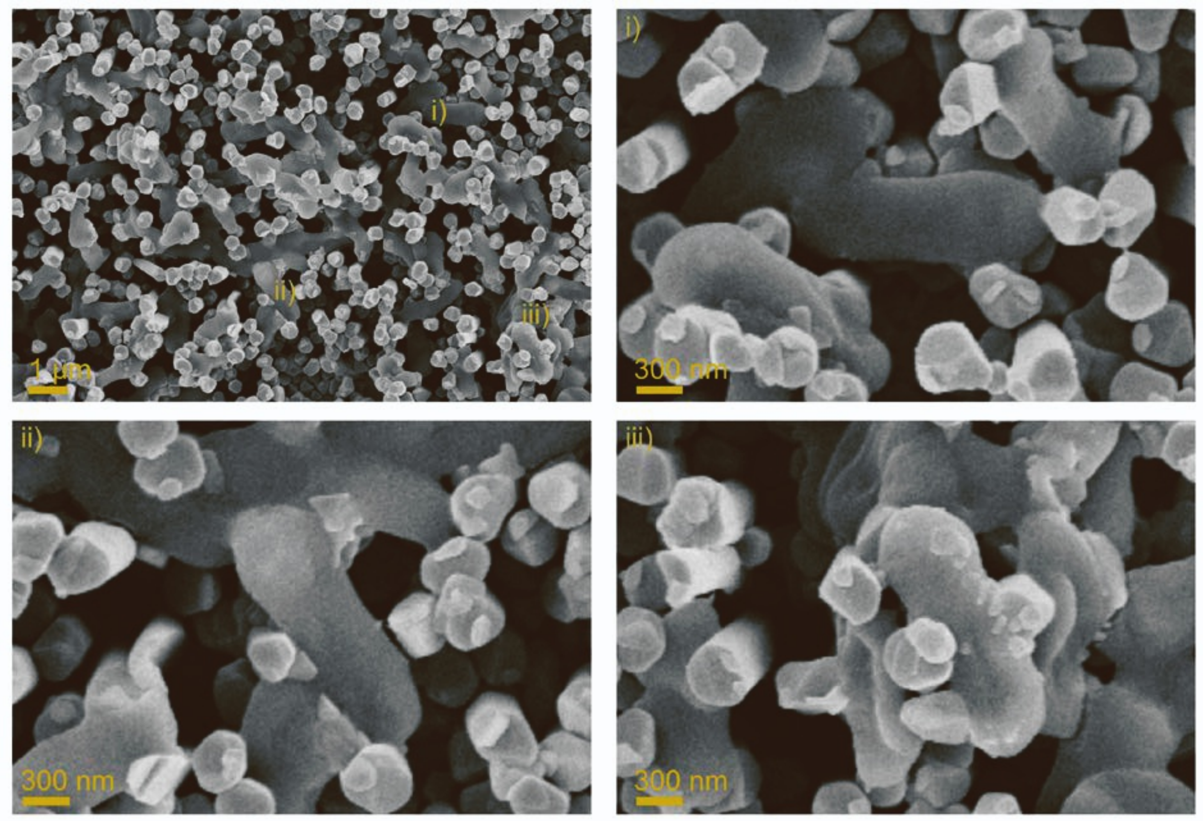

Figure S1. ***S. oneidensis*** **cells connected to silver nanorods, related to Figure 2.** SEM images of *S. oneidensis* WT cells loaded on an AAO silver nanostructured electrode. An overview of multiple cells loaded on the Ag nanostructured surface is shown in top left with annotated highlights for cells with intact cell membrane. High magnification images of the cells are shown with the connected annotation.

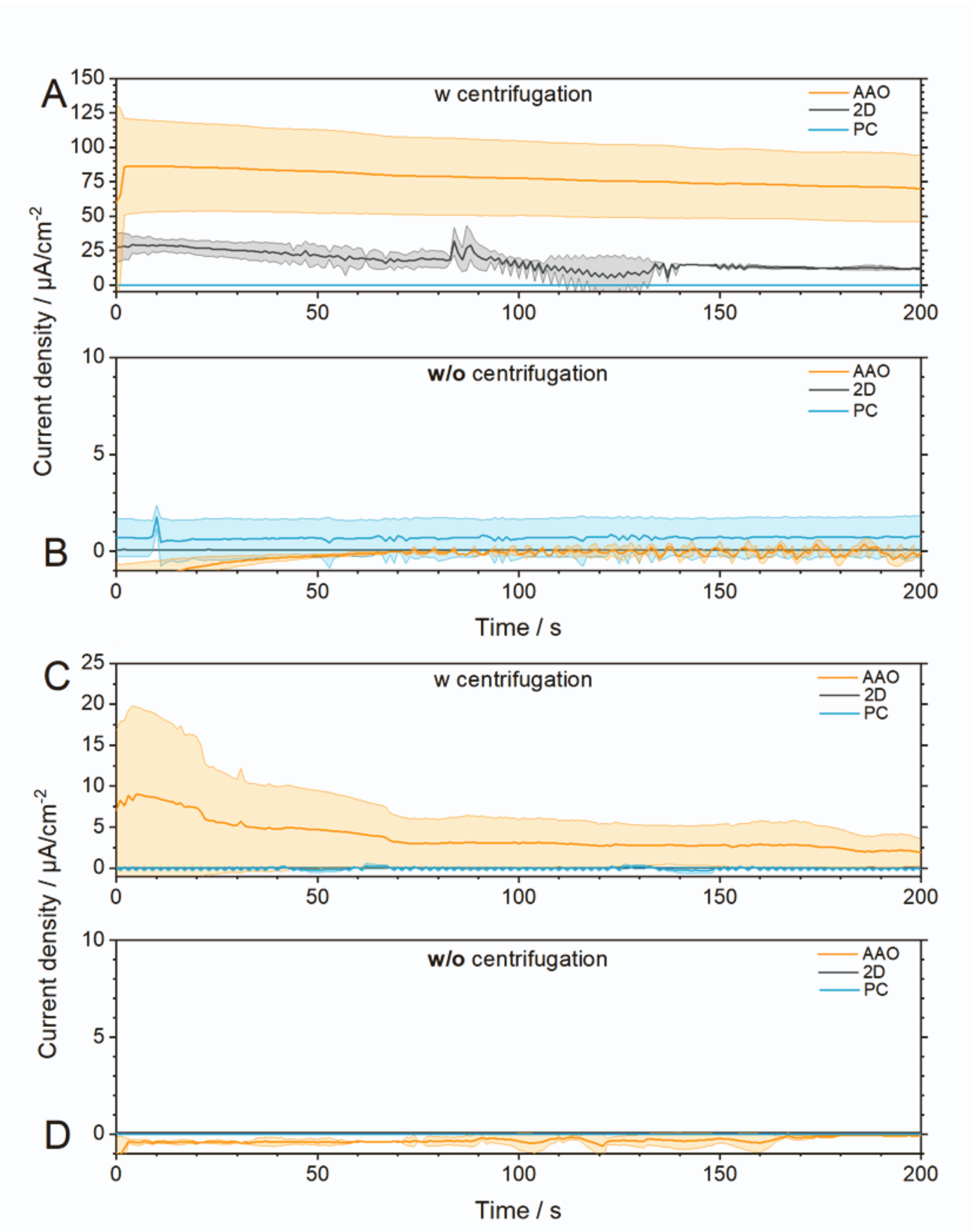

Figure S2. Current profiles for the chronoamperometry analysis, related to Figure 4. Showing *S. oneidensis* (A-B) WT and (C-D) dOMC strains on planar (2D) and nanostructured electrodes (AAO and PC). Chronoamperometry analysis was performed (A and C) with and (B and D) without the application of centrifugation prior to the bioelectrochemical analysis.

Table S1. Composition of LB1 medium, related to STAR Methods.

Components	Concentration
Tryptone	10 g/L
Yeast extract	5 g/L
NaCl	5 g/L

pH was adjusted to 7.2

Table S2. Composition of LB2 medium, related to STAR Methods.

Components	Concentration
Tryptone	10 g/L
Yeast extract	5 g/L
NaCl	5 g/L
HEPES	50 mM
Fumarate	50 mM
Lactate	50 mM

pH was adjusted to 7.2

Table S3. Composition of M4 medium, related to STAR Methods.

Components	Concentration	
K_2HPO_4	0.22 g/L	
KH_2PO_4	0.09 g/L	
HEPES	1.41 g/L	
$NaHCO_3$	0.17 g/L	
$(NH4)_2SO_4$	1.19 g/L	
NaCl	8.77 g/L	
$MgSO_4$	1 mM	
$CaCl_2$	0.1 mM	
Casamino acids	1 g/L	
Lactate	50 mM	
Trace elements:		
$CoCl_2$	11.9 mg/L	
$CuSO_4$ x 5 H_2O	0.5 mg/L	
H_3BO_3	35.1 mg/L	
Fe(II)Cl_2 x 4 H_2O	10.74 mg/L	
Na_2EDTA	250 mg/L	
$MnSO_4$ x 1 H_2O	2.13 mg/L	
Na_2MoO_4 x 2 H_2O	9.36 mg/L	
Na_2SeO_4 x 6 H_2O	2.83 mg/L	
NaCl	5.84 mg/L	
$NiCl_2$ x 6 H_2O	11.9 mg/L	
$ZnSO_4$ x 7 H_2O	3 mg/L	

pH was adjusted to 7.2

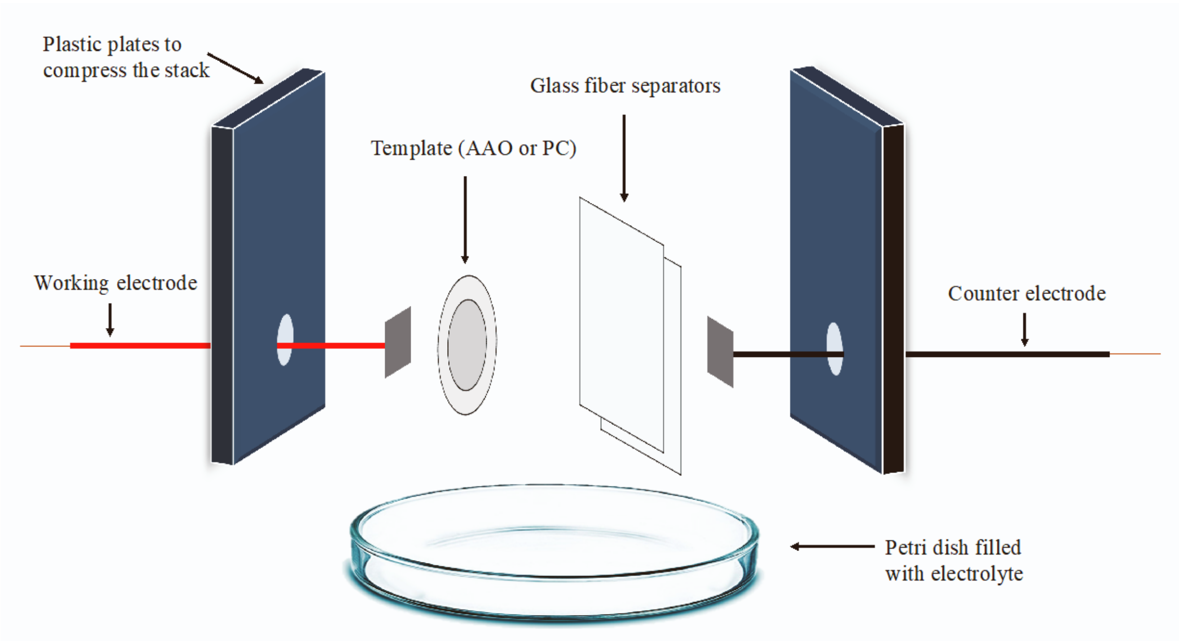

Figure S3. Electrodeposition setup, related to STAR Methods. Schematic representation of the template-assisted electrodeposition cell used to produce Ag nanostructured electrodes.

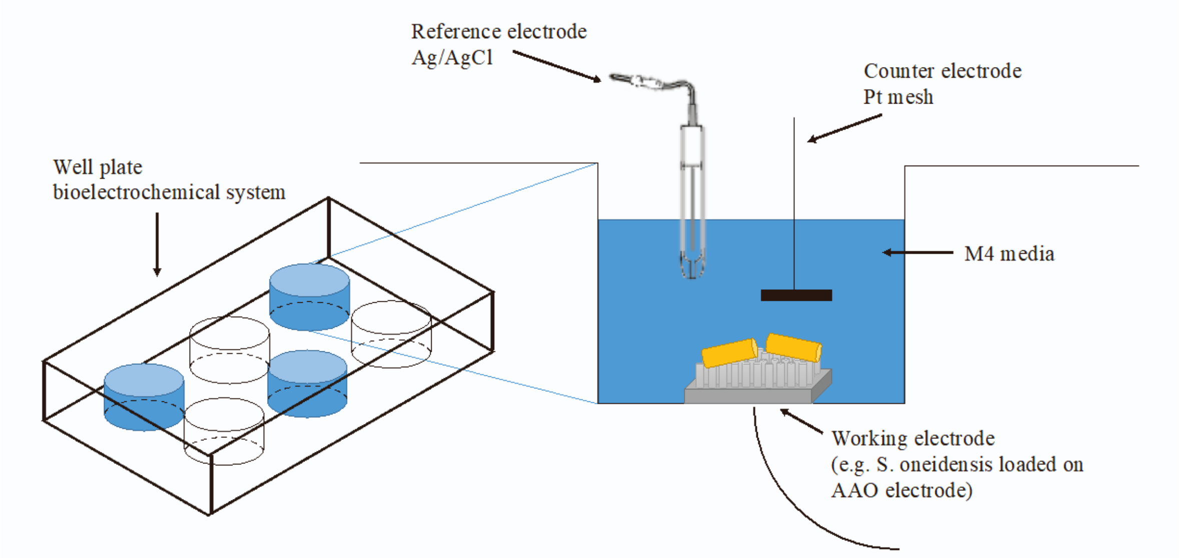

Figure S4. Customized bioelectrochemical system, related to STAR Methods. Schematic representation of the customized well plate bioelectrochemical systems developed in this study and used for the bioelectrochemical analysis (i.e. LSV and CA). Each well plate contains a complete triplicate of BESs.
